# An Incremental Learning Framework to Enhance Teaching by Demonstration Based on Multimodal Sensor Fusion

**DOI:** 10.3389/fnbot.2020.00055

**Published:** 2020-08-27

**Authors:** Jie Li, Junpei Zhong, Jingfeng Yang, Chenguang Yang

**Affiliations:** ^1^Key Laboratory of Autonomous Systems and Networked Control, School of Automation Science and Engineering, South China University of Technology, Guangzhou, China; ^2^School of Science and Technology, Nottingham Trent University, Nottingham, United Kingdom; ^3^Shenyang Institute of Automation Guangzhou Chinese Academy of Sciences, Guangzhou, China; ^4^Bristol Robotics Laboratory, University of the West of England, Bristol, United Kingdom

**Keywords:** incremental learning network, teaching by demonstration, teleoperation, data fusion, robot learning

## Abstract

Though a robot can reproduce the demonstration trajectory from a human demonstrator by teleoperation, there is a certain error between the reproduced trajectory and the desired trajectory. To minimize this error, we propose a multimodal incremental learning framework based on a teleoperation strategy that can enable the robot to reproduce the demonstration task accurately. The multimodal demonstration data are collected from two different kinds of sensors in the demonstration phase. Then, the Kalman filter (KF) and dynamic time warping (DTW) algorithms are used to preprocessing the data for the multiple sensor signals. The KF algorithm is mainly used to fuse sensor data of different modalities, and the DTW algorithm is used to align the data in the same timeline. The preprocessed demonstration data are further trained and learned by the incremental learning network and sent to a Baxter robot for reproducing the task demonstrated by the human. Comparative experiments have been performed to verify the effectiveness of the proposed framework.

## Introduction

With the development of control theory and sensor technology, robots have been widely applied in various fields, especially in industry and social service. It plays an increasingly vital role in human daily life, such as entertainment, education, and home service, etc. In most cases (Billard et al., 2008; Yang et al., 2018; Fang et al., 2019), robots need to learn and execute many complex and repetitive tasks, which include learning the motion skills from observing humans performing these tasks, also known as teaching by demonstration (TbD). TbD is an efficient approach to reduce the complexity of teaching a robot to perform new tasks (Billard et al., 2008; Yang et al., 2018). With this approach, a human tutor demonstrates how to implement a task to a robot easily (Ewerton et al., 2019). Then, the robot learns the key features from human demonstration and repeats it by itself. Obviously, the main issue of robot learning is how to learn more critical features from the demonstration to fulfill a certain task well. Therefore, it is essential to take account of some learning methods to learn much more useful features effectively. In this sense, robot learning contains two tasks: motion perception based on multiple sensors and features learning with efficient methods. Different modalities of sensors can enable obtaining an accurate description of the target motions and enrich the information (Chavez-Garcia and Aycard, 2016), and the learning methods promote to learn the desirable features. In this paper, we developed a novel robot learning framework to enhance the performance of TbD. This framework combines the superiority of the incremental learning network and the multiple sensors fusion.

Multimodal sensor fusion is a promising technique to create more accurate, more complete, or more dependable data with less uncertainty (Elmenreich, 2002; Haghighat et al., 2011) to enrich the features of demonstration data. Compared with individual sensor data, the multi-sensor fusion data have a distinctive preponderance in four general aspects (Mitchell, 2007). First, a higher resolution and richer semantic become possible for usage in the representation of the data. Second, the fused sensory data or data from disparate sources can reduce the uncertainty of information than these sources are used individually. Besides, a more all-sided view regarding the object is allowed for a coherent space to enhance the completeness of the information. Last, if the data are noisy or have errors, the fusion process will reduce or eliminate noise and errors. Hence, the data through the fusion process are possible to achieve the desired result with enhanced reliability, extended parameter coverage, and improved resolution (Fung et al., 2017). The system with multiple sensors provides immense opportunities for applications in a wide variety of areas. Applications that benefit from the sensor fusion technology cover many engineering fields which include internet of things (Din et al., 2015; Bijarbooneh et al., 2016), automation systems (Iyengar et al., 2003; Caterina et al., 2015), computer vision (Eitel et al., 2015), target tracking (Smith and Singh, 2006), health care (Medjahed et al., 2011; Koshmak et al., 2016), mechatronics (Luo and Chang, 2012), and robotics (Chung et al., 2011).

Recently, the multimodal sensor fusion is widely engaged in human–robot interaction (HRI) to enhance the performance of interaction (Gui et al., 2017; Argyrou et al., 2018; Deng et al., 2018; Fang et al., 2019; Li C. et al., 2019). Gui et al. (2017) designed a multimodal rehabilitation HRI system, which combines the electroencephalogram (EEG)-based HRI and electromyography (EMG)-based HRI to assistant gait pattern, to enhance active participation of users for gait rehabilitation and to accomplish abundant locomotion modes for the exoskeleton. Argyrou et al. (2018) proposed a human–robot collaborative monitoring system that can fuse data from multiple sources to estimate the execution status of the tasks more accurately. Deng et al. (2018) proposed an improved HRI by fusing the operator's gesture and speech to control the movements of a robot. The fusion of gesture and speech improved the accuracy, efficiency, and naturalness of the proposed system. Li C. et al. (2019) developed an augmented reality interface based on HRI that the Kalman filter (KF) algorithm was used to fuse the position and velocity signals from the Leap Motion sensor and the Kinect sensor to improve the tracking performance, aiming to provide an easier and accurate interaction. Wan et al. (2017) developed an intelligent system to teach robots to do object assembly through multimodal vision for next-generation industrial assembly. Zeng et al. (2019) proposed a TbD system to teach the robots to learn specific tasks based on multiple sensor fusion. Compared with single modal data, the multimodal data provide a more rich and complementary information source to facilitate the diversity of robot TbD. These applications benefit from sensor fusion technology because of multi-sensor-based data fusion algorithms. Due to the varieties of the nature of the fusion process, different algorithms are used to enable the different levels of sensor fusion, such as KF (Kalman, 1960), support vector machine (SVM) (Cortes and Vapnik, 1995; Waske and Benediktsson, 2007), particle filter (Crisan and Doucet, 2002), Bayesian inference method (Khaleghi et al., 2013), fuzzy sensor fusion approach (Gibson et al., 1994), and artificial neural network (Hu, 2010), etc. Studies showed that the KF is ideally suited to coping with multi-sensor estimation and data fusion problems. This is mainly because the algorithm runs best with well-defined state descriptions (such as positions, velocities) and for states where observation and time-propagation models are also well-understood. In this paper, the KF is used to fuse the positions and velocities of a humanoid robot to achieve an overall complete description of the joint positions with high accuracy and fewer uncertainties.

Sensor fusion can enable to obtain more accurate demonstration data, while effective learning methods can learn more desired features of data. A deep learning neural network, as a kind of popular feature learning algorithm, has been successfully applied in various fields because of its powerful approximation capability (Ciresan et al., 2012; Marblestone et al., 2016; Sze et al., 2017). Although this advantage makes it apply in amounts of areas, it often needs a large number of datasets to train the network. Due to this, a complicated network structure is needed to deal with them, and then the network will suffer from a time-consuming process. Apart from that, the network is also faced with the issue that entire retraining when new samples are inputted. Considering these problems of deep structure learning methods, Chen and Liu (2017) proposed an incremental learning method, which provides an alternative way for deep structure neural network (Liu and Chen, 2017). The incremental learning network can rapidly learn and model the target system without a retraining process if new samples are fed into it. Also, the structure of this network can be expanded flexibly in a wide sense. Like a deep structure neural network, the approximation capability of an incremental learning network is universal (Chen et al., 2018). Hence, it has been successfully engaged in different fields employing efficient modeling and fast learning ability. These applications mainly involved two aspects: classification and regression. Most researchers employ this algorithm in various kinds of classification (Zhang et al., 2018; Li J. et al., 2019). For example, Zhang et al. (2018) applied it to recognize facial expression to improve the accuracy of recognition. Based on this method, Wang et al. (2018) integrated it with the convolution neural network to classify EEG emotion which achieves the highest average recognition accuracy. The applications, which are involved in different curves fitting, were seldom. Luo et al. (2019) used it to estimate human intention by predicting the force of human hand. Chen et al. (2018) have proved that compared with function approximation and time series prediction, the incremental learning algorithm is superior to other learning algorithms, such as SVM, least squared SVM, and extreme learning machine (ELM), on regression performance. It is noted that the incremental learning network and ELM algorithm (Huang et al., 2004) are similar among these methods. Both networks have the structure of a single layer. Also, both networks are thought to have potential advantages in learning rate and generalization ability (Huang et al., 2006; Chen et al., 2018). Apart from that, the incremental learning network can be employed in other scenarios, such as fault diagnosis (Zhao et al., 2019) and monkey oculomotor decision decoding (Shi et al., 2020). However, this method is seldom used in HRI to improve the performance of robot learning.

For the TbD system, we can teach a robot to move as the desired trajectory. However, human movement is not always necessarily optimal for the robot when it tries to repeat and accomplish a task. Therefore, teaching a robot remotely, there will be some deviations between the robot's trajectory and the target trajectory. Through learning based on a neural network, the robot's trajectory can approach the target trajectory. To achieve that, the incremental learning algorithm is used to learn the fused features of a certain task from different sensors to enhance the learning performance. Then, experiments are performed to verify the effectiveness of the proposed multimodal framework.

The main contribution of this paper is to develop a framework that integrates the advantages of the multiple modal information fusion with the approximation capability of the incremental learning algorithm to enhance the performance of the TbD system. The remainder of the paper is organized as follows. The System Outline section presents the whole architecture of the proposed framework. The details of the data collection, preprocessing, and learning methods are introduced in the Methodology section. The Experiments and Results section describes the experimental settings and explains the results of the experiments. The experimental results are discussed in the Discussion section. The Conclusions and Future Work section concludes this work.

## System Outline

### System Description

**Figure 1:**
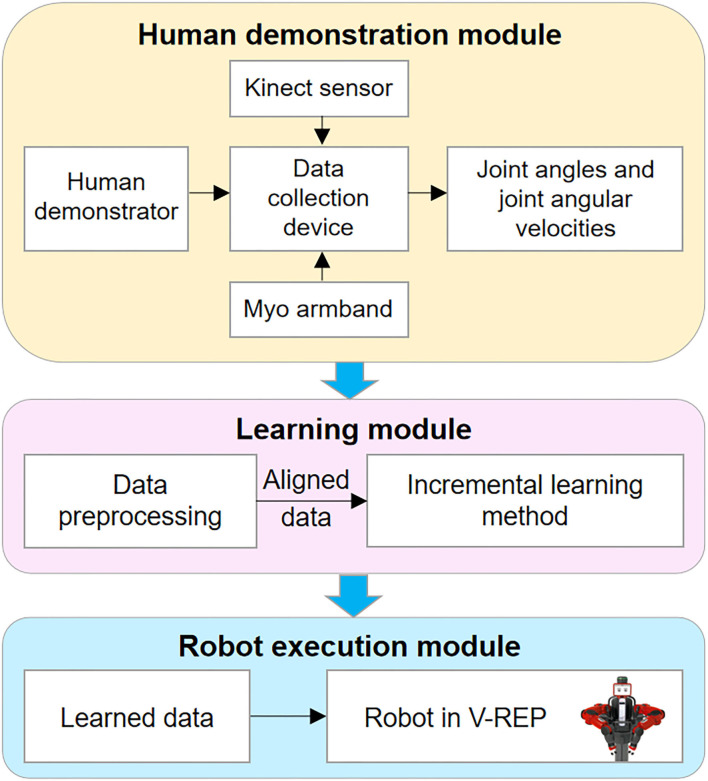
Outline of the robot teaching by demonstration (TbD) system.

The proposed framework of the TbD is shown in Figure 1, which consists of three modules: the human demonstration module, the learning module, and the robot execution module.

Human demonstration module: This module, which is a virtual demonstration system, allows the human demonstrator to control the Baxter robot in Virtual Robot Experimentation Platform (V-REP) *via* human joint information. The human joint information including joint angles and joint angular velocities is recorded by the Kinect sensor and Myo armbands separately.

The learning module: This module includes two steps: data preprocessing and incremental learning. The target of data preprocessing is to align the time series information of the demonstrated tasks in the same timeline. After that, an incremental learning method is used to learn the preprocessed data.

The robot execution module: The main function of this module enables the robot to complete the task with the learned data from the training module. To this end, a specific task will be performed by a robot to verify the effectiveness of the proposed framework.

### System Principle

**Figure 2:**
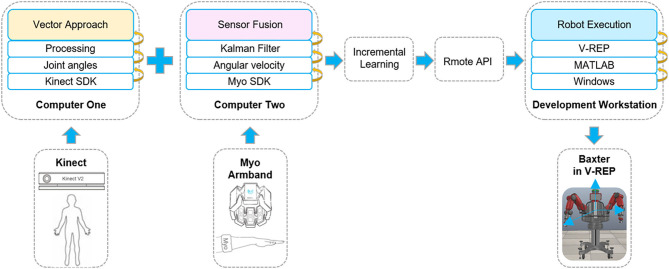
The principle of the whole system. The multimodal signals are collected by the corresponding sensors from human demonstration. Two sensors are connected to different computers. Then, through incremental learning, the demonstration data are transmitted to the Baxter robot through the remote application programming interface (API) indirectly. The Baxter robot is connected to the development workstation directly. Thus, the robot can execute the demonstrated task.

The principle of the overall system based on the proposed method with multimodal sensor data fusion is presented in Figure 2. As shown in Figure 2, it consists of a Kinect sensor, two Myo armbands, and a Baxter robot. Kinect sensor is a motion capture device which is used to capture the motion of the human body. Myo armband, as a wearable device, is used to capture the human joint angular velocities. Baxter is a versatile semi-humanoid robot which is equipped with several advanced sensing technologies (including force, position, and torque sensing) which allow it to be applied in scientific research. V-REP is a powerful open-source robot simulator with an integrated development environment, distributed control architecture, and rich user interface to make it be an ideal platform for robot simulations. The remote application programming interface (API) in V-REP can control the robot simulation from an external application or remote hardware. This work will simulate the Baxter robot and control it by two developed API clients in V-REP.

**Figure 3:**
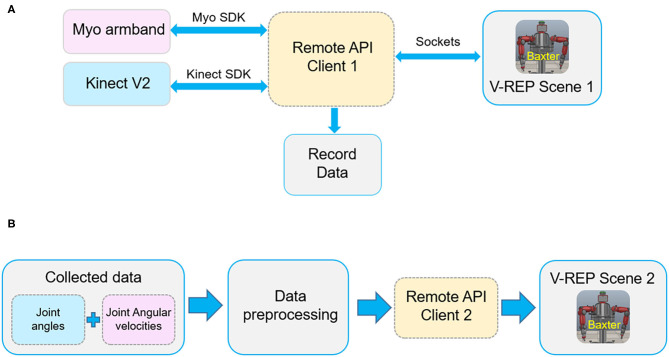
The communication links of the robot virtual demonstration system. **(A)** The communication of data collecting. **(B)** The communication of robot simulation.

Figure 3 shows the communication links of the virtual TbD system. It is noted that the data collected from the Kinect sensor and Myo armbands are separately recorded by two computers. Two sensors both can recognize human hand gestures. To capture the joint angles and angular velocities simultaneously, the hand state is used to control the start or end of the data collecting. When the human demonstrator's hand state is open, the data of joint angles and angular velocities will be recorded and saved in different files. Instead, the data collecting work will stop.

According to the designed human demonstration model, joint angles and angular velocities are recorded from the multiple demonstrations based on a specific task. Then, the raw demonstration data will be preprocessed and learned by the robot learning module. After that, the learned data are transferred to the Baxter robot in V-REP by MATLAB for execution. We can verify the effectiveness of the proposed method by the execution result of the Baxter robot.

## Methodology

The proposed incremental learning framework includes three processes: data collection, data preprocessing, and data learning method, which correspond to the three modules mentioned above. In this section, the data collection and preprocessing processes will be introduced, and the details of the incremental learning network and the multi-sensor fusion algorithm KF also will be given.

### Data Collection

In this section, we will introduce how to capture the human joint angles and angular velocities using a Kinect sensor and two Myo armbands in detail, respectively.

#### Calculation of Joint Angles Using the Space Vector Approach

Since we can get the three-dimensional (3D) joint coordinates of a human body using the Kinect sensor, the key to obtain the joint angles is how to convert these coordinates into corresponding angles. This problem can be addressed by the space vector approach. As we know, the distance between two specified 3D points *A*(*x*_*a*_, *y*_*a*_, *z*_*a*_) and *B*(*x*_*b*_, *y*_*b*_, *z*_*b*_) can be calculated by the following equation:

(1) $$\begin{array}{l} d_{AB}=\sqrt{{\left(x_{b}-x_{a}\right)}^{2}+{\left(y_{b}-y_{a}\right)}^{2}+{\left(z_{b}-z_{a}\right)}^{2}} \end{array}$$

Essentially, the distance *d*_*AB*_ is equal to the norm of the vector $\vec{AB}=\left(x_{b}-x_{a},y_{b}-y_{a},z_{b}-z_{a}\right)$. In a 3D space, the law of cosines can be used to calculate the angles between two known vectors. In the Kinect coordinate, a joint can be expressed as a vector. So, the angle between joint 1 ($\vec{PO}$) and joint 2 ($\vec{OQ}$) can be computed as:

(2) $$\begin{array}{l} \cos\left(\vec{\text{PO}},\vec{\text{OQ}}\right)=\frac{\vec{\text{PO}}\cdot \vec{\text{OQ}}}{\left|\vec{\text{PO}}|\cdot |\vec{\text{OQ}}\right|} \end{array}$$

According to Equation (1), we can transform the coordinates returned by Kinect into corresponding vectors. Then, the angles of these vectors can be calculated by Equation (2).

**Figure 4:**
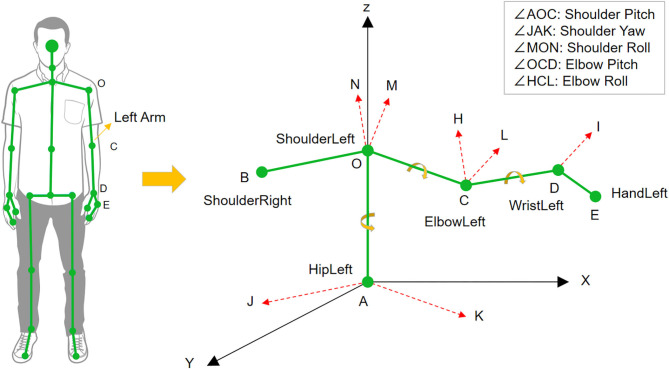
The models of human body skeleton and human left arm.

The models of the full human body and the left arm are shown in Figure 4. The coordinate system of Kinect in Cartesian space is constituted by three directed straight lines AX, AY, and AZ, where point A is the origin of the coordinate. According to Equation (2), the shoulder pitch angle ∠*AOC* can be calculated by the vectors $\vec{OA}$ and $\vec{OC}$ from the position coordinates of points *A*, *O*, and *C*. The elbow pitch angle ∠*OCD* is calculated using the same method. We can get the shoulder yaw angle ∠*JAK* in a similar way. The difference is that the vectors $\vec{AJ}$ and $\vec{AK}$ are obtained by projecting vectors $\vec{OB}$ and $\vec{OC}$ to the *XZ* plane.

To calculate the shoulder roll angle, the cross product is applied to get the normal vector of different planes. The normal vectors of the BOC and OCD planes can be calculated by:

(3) $$\begin{array}{l} \{\begin{array}{l} \vec{OM}=\vec{OB}\times \vec{OC}\\ \vec{CH}=\vec{CO}\times \vec{CD} \end{array} \end{array}$$

Then, translating the vector $\vec{CH}$ along the vector $\vec{CO}$ to point *O* can get the vector $\vec{ON}$. So, the calculation of the shoulder roll angle ∠*MON* is addressed. Using the same method, we can get elbow roll angle ∠*HCL*, which is the angle between the planes of OCD and CDE.

Here, only three joint angles of the human arm involving the human shoulder and elbow are collected.

#### Calculation of Joint Angular Velocity From Myo Armband

To obtain the joint angular velocity, two Myo armbands are needed to wear on the user's upper arm and forearm. The quaternion method is used to obtain the joint angles. Then, the joint angular velocities can be computed based the difference of the joint angles. According to Yang et al. (2018), we can assume that the joint angle of the initial position is zero. When the user's arm is moved from a pose *T* to a new pose *P*, the angle from pose *T* to *P* is the rotation angle. For the pose *P*, pose *T* can be regarded as the initial pose, and the rotation angel is the joint angle.

Assume that the Myo armband's orientation is expressed by frame (*x*_0_, *y*_0_, *z*_0_) in the initial position, the current orientation is expressed by frame (*x*_1_, *y*_1_, *z*_1_). Then, the angular velocities of the shoulder roll, shoulder yaw, and shoulder pitch can be obtained by the forearm armbands. The velocities of the elbow roll and pitch angles are acquired by the armbands worn on the upper arm. Thus, five joint angular velocities are obtained for each arm from a pair of Myo armbands.

Thus, we can obtain two different modalities information of human arm. After that, these joint angles and the joint angular velocities will be fused by the KF algorithm.

### Data Preprocessing

The demonstration data from the Kinect sensor and Myo armband will be preprocessed before they are fed into the incremental learning method. Firstly, the data fusion method based on the KF is used to fuse the joint angles and joint angular velocities to obtain a more accurate and smooth dataset. Since the demonstration data are not matched in the timeline, then the dynamic time warping (DTW) algorithm is applied to align them. Here, the two preprocessing methods will be introduced briefly.

#### Data Fusion by Kalman Filter

KF, as one of the most powerful sensor fusion algorithms, can smooth noisy input data and optimize the estimation of the current state based on current measurements and the previously estimated state. These current measurements are often multiple sequential measurements from several sensors with noise. The existing works have proved that the estimate of the system's state from multiple sensors is better than the estimate obtained from only one sensor (Gui et al., 2017). Therefore, the sensor fusion based on the KF is used to improve the accuracy of data.

This algorithm uses a series of state prediction and measurement update steps to update the state of the target object. The prediction and update steps are presented below. For a continuous simplified linear system, the dynamic model is described as follows (Davari et al., 2016):

(4) $$\begin{array}{l} \begin{array}{l} ẋ\left(t\right)=Fx\left(t\right)+Gu\left(t\right)+Mn\left(t\right)\\ z\left(t\right)=Hx\left(t\right)+v\left(t\right) \end{array} \end{array}$$

where *x*(*t*) ∈ ℝ^*n*^ is the state vector, *u*(*t*) ∈ ℝ^*m*^ is the deterministic input vector, *z*(*t*) ∈ ℝ^*p*^ is the measurement vector, *n*(*t*) ∈ ℝ^*q*^ is the white noise term for the state vector with zero-mean and covariance ***S***, and *v*(*t*) ∈ ℝ^*p*^ is the noise term for measurement vector with zero-mean and covariance ***R***. ***F*** ∈ ℝ^*n*×*n*^ and ***G*** ∈ ℝ^*n*×*n*^ are both system matrices. ***M*** and ***H*** are parameter matrices related to the noise and measurement, respectively. The KF model of the linear system can be expressed by the following equations (Simon, 2006):

(5) $$\begin{array}{c} \dot{\hat{x}}\left(t\right)=F\hat{x}(t)+Gu\left(t\right)+K\left(t\right)\left[z\left(t\right)-H\hat{x}\left(t\right)\right]\;\\ \begin{array}{c} K\left(t\right)=\Sigma \left(t\right)H^{T}R^{-1}\;\\ \dot{\Sigma }\left(t\right)=F\Sigma \left(t\right)+\Sigma \left(t\right)F^{T}+MSM^{T}-\Sigma \left(t\right)H^{T}R^{-1}H\Sigma \left(t\right) \end{array} \end{array}$$

where ***K***(*t*) is the filter gain, $\dot{\hat{x}}\left(t\right)$ is the state estimation of *x*, and **Σ**(*t*) is the estimation of covariance.

For the above equations, we assume that *x*(0), *n*, and *v* are uncorrelated to each other, and all the KF parameters are first order. If each joint of human arm is considered separately, we have ***F*** = 0, ***G*** = 1, ***M*** = 1, and ***H*** = 1. Thus, Equations (4, 5) can be simplified as:

(6) $$\begin{array}{l} \begin{array}{l} {ẋ}_{i}=u_{i}+n_{i}\\ z_{i}=x_{i}+v_{i} \end{array} \end{array}$$

(7) $$\begin{array}{l} \begin{array}{l} {\dot{\hat{x}}}_{i}=u_{i}+K\left(t\right)\left[z_{i}-{\hat{x}}_{i}\right]\;\\ K=\Sigma R^{-1}\\ \dot{\Sigma }=S-\Sigma R^{-1}\Sigma \end{array} \end{array}$$

where *u*_*i*_ is the *i*th joint angular velocity of the human arm, *z*_*i*_ is the *i*th joint position (or the joint angles), and ${\dot{\hat{x}}}_{i}$ is the fused data of the *i*th joint. Note that the parameters *K*, Σ, *R*, and *S* are scalar values.

#### Data Preprocessing With Dynamic Time Warping

Through the human demonstration module, the angles and angular velocities of the human joints are collected from multiple demonstrations. As aforementioned, the time for every demonstration is not the same. We employ the DTW algorithm to align them in the same timeline.

DTW is a method to measure the similarity of two time series with different lengths. It has been widely used in processing the temporal sequences of video, audio, and graphics data. If two given temporal sequences *g* and *k* satisfy the boundary, monotonicity, and step size conditions, the objective of DTW can be transformed into the optimal match path problem between the two sequences. We expressed this optimal match path as:

(8) $$\begin{array}{l} DTW\;\left(y_{1},y_{2}\right)=\min\left(d\left(y_{1},y_{2}\right)\right) \end{array}$$

where *d*(*y*_1_, *y*_2_) represent the distance between sequences *y*_1_ and *y*_2_. Then, the Dynamic programming is used to solve Equation (8). At the same time, an accumulated cost matrix *E* with the dimension of *m* × *n* is generated. The expression of matrix ***E*** is written as follows:

(9) $$\begin{array}{l} E\;\left(l_{1},l_{2}\right)=\left(y_{1},y_{2}\right)+\{\begin{array}{l} \;0\text{ if }l_{1}=1\text{ and }l_{2}=1\;\\ E\left(l_{1},l_{2}-1\right)\text{ else if }l_{1}=1\text{ and }l_{2}>1\\ E\left(l_{1}-1,l_{2}\right)\text{ else if }l_{1}>1\text{ and }l_{2}=1\\ \min(E\left(l_{1},l_{2}-1\right),\\ \;E\left(l_{1}-1,l_{2}\right),\\ \;E\left(l_{1}-1,l_{2}-1\right))\text{ otherwise} \end{array} \end{array}$$

where *l*_1_ and *l*_2_ are the length of the sequences *y*_1_ and *y*_2_, respectively.

### Incremental Learning Method

**Figure 5:**
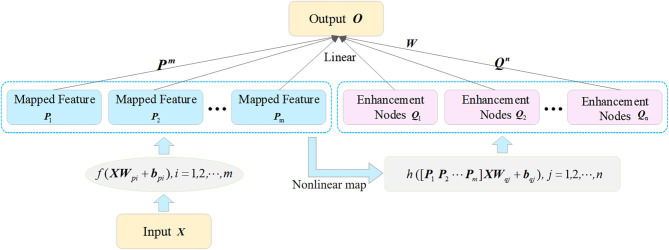
The architecture of incremental learning network.

The incremental learning algorithm is essentially a single-layer neural network that the structure can be dynamically expanded in a wide sense. It is constructed based on the random vector functional link neural network (FLNN). The architecture of the incremental learning network is shown in Figure 5.

The input of this network is composed of two parts: the mapped features and the enhancement nodes. As shown in Figure 5, the original inputs are first transformed into a group of mapped features to extract the random features by some linear feature mappings. Then, the mapped features are extended to enhancement nodes by non-linear mappings. Further, the mapping features and the enhancement nodes in the input layer are both connected with the output linearly. Thus, the weights between the input layer and the output layer can be calculated by the ridge regression of the pseudo-inverse method.

The detailed process of the incremental network is presented as follows. For a given input dataset {***X***} and *m* feature mapping function ***f***_*i*_, *i* = 1, 2, …, *m*, the *i*th mapped features can be calculated as:

(10) $$\begin{array}{l} P_{i}=f_{i}\left({XW}_{p_{i}}+b_{p_{i}}\right),\;i=1,2,\cdots \;,m \end{array}$$

where ***X*** ∈ ℝ^*m*×*n*^, *m* is the number of training samples; *n* is the size of each training sample; both the bias unit ***b***_*p*_*i*__ and the weights ***W***_*p*_*i*__, which connect the original input and the mapped features, are randomly generated. It is noted that the functions ***f***_*i*_ and ***f***_***l***_ are equal for *i* ≠ *l*. We denote the first *i*th groups of mapped features as $P^{i}\equiv \left[P_{1}\;P_{2}\;{\cdots \;P}_{i}\right]$ and express the non-linear mappings connected the mapped features with enhancement nodes as ***h***_*j*_, *j* = 1, 2, …, *n*. Then, using the non-linear function ***h***_*j*_, the relationship between the mapped features ***P***_*i*_ and ***Q***_*j*_, the enhancement nodes can be built. The *j*th group of enhancement nodes is expressed as:

(11) $$\begin{array}{l} Q_{j}=h_{j}\left(P^{m}W_{p_{j}}+b_{p_{j}}\right),\;j=1,2,\cdots \;,n \end{array}$$

where ***W***_*p*_*j*__ and ***b***_*p*_*j*__ are randomly generated, and ***W***_*p*_*j*__ are the weights connecting the mapped features and the enhancement nodes. Likewise, the first *j*th group of enhancement nodes is denoted as $Q^{j}\equiv \left[Q_{1}\;Q_{2}\;{\cdots \;Q}_{j}\right]$. The enhancement nodes ***P***_***i***_ together with the mapped features *Q*_*j*_ form the actual input of the incremental learning network $A\equiv \left[P_{1},\;{\cdots \;,\;P}_{m}{,Q}_{1},\;{\cdots \;,\;Q}_{n}\right]=\left[P^{m}\;Q^{n}\right]$. Hence, the output *O* of this network is computed as:

(12) $$\begin{array}{l} O={AW}_{m}^{n}, \end{array}$$

where the weights $W_{m}^{n}$ connect the output layer and the input layer. Since the target output *O* is given, we can calculate the weights $W_{m}^{n}$ as follows:

(13) $$\begin{array}{l} W_{m}^{n}=A^{+}O, \end{array}$$

Here, the rigid regression learning algorithm is used to solve the pseudo-inverse ***A*** in Equation (13). According to this algorithm, the pseudo-inverse ***A*** is obtained by the following equation:

(14) $$A=\underset{\lambda \to 0}{lim}{\left(\lambda I+AA^{T}\right)}^{-1}A^{T},$$

Algorithm 1 presents the whole training process of the incremental learning network.

**Algorithm 1:** The procedure of the incremental learning network.

**Input**: Demonstration dataset ***X***, mapped feature group *m*, and enhancement nodes group *n*.
**Output**: The parameter matrix ***W***.
**for** *i* = 1 to *m* do
Randomly initialize the weights ***W***_***p***_***i***__ and bias unit ***b***_***p***_***i***__;
Calculate ***P***_***i***_ according to Equation (10).
**end**
Set mapped features group $P^{m}\equiv \left[P_{1}\;P_{2}\;{\cdots \;P}_{m}\right]$;
**for** *j* = 1 to *n* do
Randomly initialize the weights ***W***_***p***_***j***__ and bias unit ***b***_***p***_***j***__;
Calculate ***Q***_***j***_ according to Equation (11).
**end**
Set the enhancement nodes group $Q^{n}\equiv \left[Q_{1}\;Q_{2}\;{\cdots \;Q}_{n}\right]$;
Calculate weights $W_{m}^{n}$ according to Equation (13).

As aforementioned, the ELM method and the incremental learning method both are single-layer neural networks, and the learning speed of two methods is also fast. For the incremental learning network, if the learning cannot reach the desired result, it can be addressed by inserting additional enhancement nodes in a wide sense not deep way to achieve a better performance. The increase of the enhancement nodes will result in the recalculation of weights. It is worth noting that only a part of the weights needs to be recalculated, not all weights. The new weights are calculated by the following equations:

(15) $$\begin{array}{l} W_{m}^{n+1}=\left[\begin{array}{l} W_{m}^{n}-{DB}^{T}O\\ O \end{array}\right], \end{array}$$

where $C=h_{n+1}\left(P^{m}W_{p_{n+1}}+b_{p_{n+1}}\right)-A^{n}D$, $D={\left(A^{n}\right)}^{+}h_{n+1}\left(P^{m}W_{p_{n+1}}+b_{p_{n+1}}\right)$, and

(16) $$\begin{array}{l} B^{T}=\{\begin{array}{l} {\left(C\right)}^{+}\;if\;C\neq 0\\ \left(1+D^{T}D\right)B^{T}{\left(A^{n}\right)}^{+}\;if\;C=0 \end{array}\;, \end{array}$$

Note that **0** is zero matrix, and ***O*** is the output of the network.

For the ELM network, the solution to improve performance is to increase the number of hidden layer neurons, which results in more connecting parameters. Thus, a great number of parameters including all weights need to be updated. It means that the ELM network suffers from a complete relearning process. In this respect, the incremental learning network is different. Besides, the incremental learning network is allowed to increase the number of input samples without relearning all samples. Likewise, only the newly added samples need to be learned by the incremental learning network. It also implies that the incremental learning network can adapt to new data without forgetting its existing knowledge, instead of relearning all samples. This is the difference in the structural expansion between the two networks.

Furthermore, the mapped features of the incremental learning network are randomly generated from the original input dataset {***X***}. In other words, the mapped features are the results of feature representation for the original input data. Feature representation can capture the efficient characteristics of the data to achieve outstanding performance in supervised learning tasks (Chen et al., 2018). It explains why the incremental learning network can learn the desired features. Also, it shows that the actual input data of the two networks are different. This implies the difference between the two networks from another aspect.

As stated above, the motivation to use the incremental learning algorithm is its convenience in a specific scene and feature learning ability.

## Experiments and Results

### Experimental Setup

**Figure 6:**
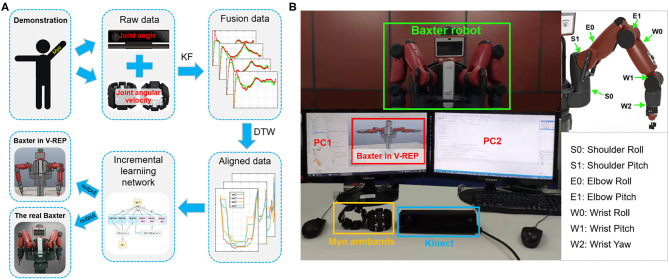
The experimental system. **(A)** The diagram of the experimental system. **(B)** The experimental platform of the demonstration phase. During demonstration, the joint angles and joint angular velocities of the human arm are collected simultaneously by Kinect and Myo armband. Then, the raw demonstration data will be fused and aligned in the same timeline by the Kalman filter (KF) and dynamic time warping (DTW) algorithms in turn. After that, the incremental learning network is applied to learn the processed data. During the robot learning phase, the learned data are directly sent to the robot model in Virtual Robot Experimentation Platform (V-REP) and the real Baxter robot.

We test our method on the Baxter robot. The experimental system is shown in Figure 6. The hardware devices consist of a Baxter robot, a Kinect sensor, and two Myo armbands. Based on the platform, two tasks (wiping and pushing) are performed to verify the effectiveness of the proposed TbD system.

In the wiping task, the robot in V-REP follows human motion to raise his left arm, move toward the left, and then put it down along the path it passed. The difficulty of this task is that the trajectories of up and down motions should be consistent. The repetitive processes with the same task are performed more than 16 times.

The wiping task is performed under the following three conditions:

- *Condition 1: with Kinect sensor data and incremental learning method*. The demonstration data are only collected from the Kinect sensor but without Myo armbands. Through processing of DTW, the incremental learning network is used to learn it. There is no data fusion in this condition.
- *Condition 2: with two sensors data (Kinect and Myo armband) and incremental learning method*. The demonstration data are collected from both Kinect and Myo armbands. In this case, the sensor fusion process is added before data preprocessing with DTW algorithm. Later, the preprocessed data are learned by the incremental learning network.
- *Condition 3: with two sensors data (Kinect and Myo) and ELM algorithm*. The demonstration data collection and processing processes are the same as the second condition. The difference is that the learning method of these data is ELM algorithm (Huang et al., 2004) instead of the incremental learning network.

In summary, the first condition is to show the performance of incremental learning network with only joint angle information but without joint angular velocities. The second condition is set to validate the proposed incremental learning framework with sensor fusion, while the third condition is to test the performance of the ELM network with sensor fusion.

To find the optimal number of feature mapping group *m* and enhancement nodes group *n*, we change *m* and *n* from 1 to 50 for the incremental learning network. The result shows that the highest accuracy appears when *m* and *n* are 6 and 8, respectively. A similar test is conducted for the ELM algorithm. We can get that when the number of hidden layer neurons is 11, the ELM network has the best accuracy.

### Experimental Results

#### Results of the Wiping Task

**Figure 7:**
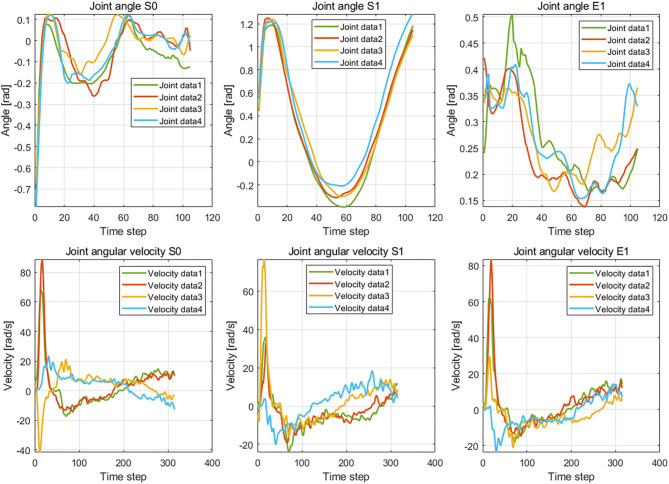
The demonstration data of joint angles and joint velocities regarding the joints S0, S1, and E1.

**Figure 8:**
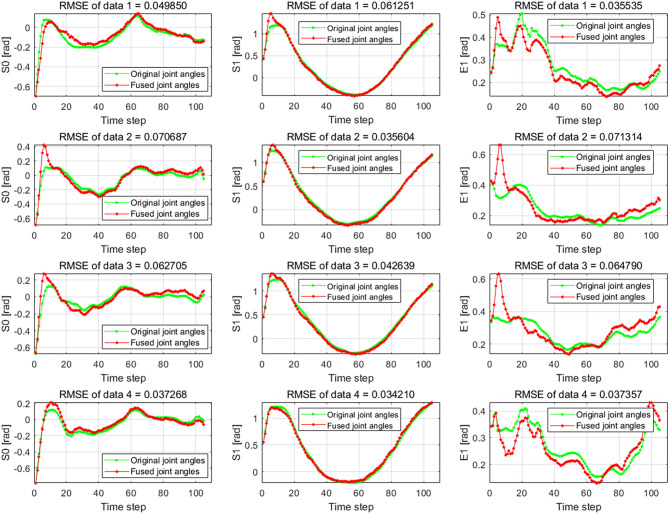
The raw data and the fused data of joint angles S0, S1, and E1. The green lines are the raw joint angles, and the red lines are the fused data by Kalman filter (KF) algorithm which fuses the joint angles and joint angular velocities.

**Figure 9:**
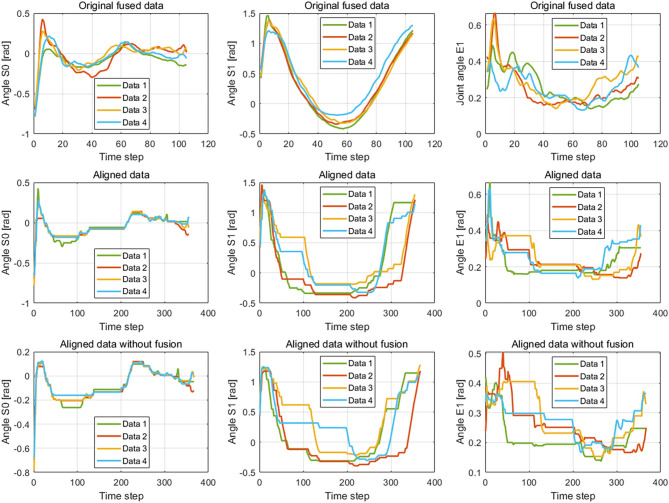
The fused data and the aligned data by the dynamic time warping (DTW) algorithm. The figures in the first line and second line show the result of the raw demonstration data fused by the Kalman filter (KF) method and alignment by the DTW algorithm, respectively. The images in the third line display the aligned result of the data collected by the Kinect sensor. The three columns are the corresponding results of joints S0, S1, and E1, respectively.

**Figure 10:**
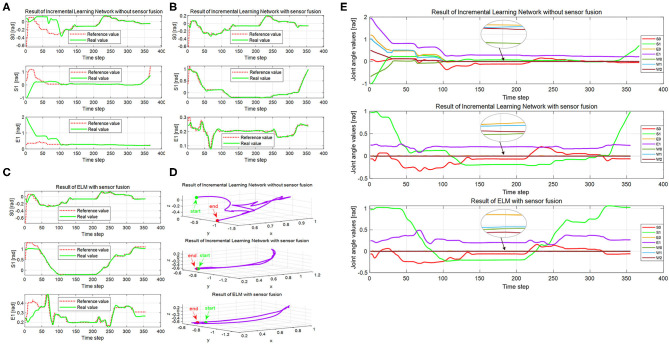
The execution results of the real Baxter robot. **(A–C)** The joint angles of the real Baxter robot under conditions 1, 2, and 3, respectively. **(D)** The trajectories of Baxter end effector under the three conditions. **(E)** Seven joint angles of the real Baxter robot under the three conditions. In panels **(A–C)**, the red dotted line is the output of the network under the three conditions, and the green solid line is the real Baxter robot's joint angles. The green solid dots are the start point of the Baxter end effector, and the red solid dots are the end point in panel **(D)**. The red, green, and purple solid lines, respectively, display the joint angles of S0, S1, and E1 in panel **(E)**, and the other colored solid lines display the rest of the four joint angles.

The results of the wiping tasks are shown in Figures 7–10. In the demonstration phase, the raw multimodal data are recorded by different sensors. Figure 7 presents four randomly selected samples of human demonstrations. The results of preprocessing are shown in Figures 8, 9. Figure 8 shows the curves of the fusion datasets. Note that there are deviations between the raw joint angles and the fused data. Figure 9 displays the aligned results of the fused datasets. Compared with the demonstration data without alignment in the timescale, the aligned data also retain the primary characteristics through the aligning process by DTW algorithm. The aligned results prepare for the next training and learning of the neural network.

The difference between the first and second conditions is that the demonstration samples are different for the incremental learning network. Since deviations between the raw original joint angles and the fusion joint angles exist, the results of DTW aligning will be different. We can observe it from the images of the second and third rows in Figure 9. The dimension of the original raw demonstration data is 105. After processing by DTW, the dimensions of these datasets are 367 and 355 for the raw data and fusion data, respectively.

The trajectories learned by the incremental learning network and ELM network are shown in Figures 10A–C with red dotted lines. For the Baxter robot, all changes of seven joint angles are aimed to obtain a desired trajectory of the end effector because the robot execution eventually depends on the end effector. We recorded the trajectory of the end effector in Cartesian space during robot execution, which is shown in Figure 10D. Seven joint angles of the real Baxter's left arm are also recorded and shown in Figure 10E.

Based on the learned results by the incremental learning network, the Baxter robot can implement the wiping task. The robot implementation includes a simulation experiment of the Baxter robot in V-REP and an experiment for real Baxter robot. And the wiping task covers four directions of continuous and smooth movement: up, down, right, and left.

#### Results of the Pushing Task

**Figure 11:**
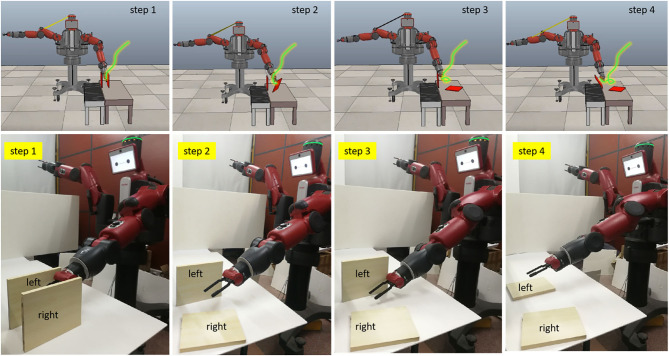
The execution results of the pushing task. The green lines in the four figures are the trajectories of the real Baxter robot. The trajectories of the real Baxter robot are sent to the Baxter model in the Virtual Robot Experimentation Platform (V-REP), which is plotted out with green lines.

To test the generalization ability of the proposed method, a pushing task is performed. The pushing task requires the robot to push two square workpieces over on the desk in sequence. In other words, the robot should firstly push the workpiece on the right to the desk. During pushing, the robot cannot touch the workpiece on the left. Then, the robot pushes the right one to the desk. The short distance between the two workpieces makes it more difficult for the robot to complete this task. Because the aims of the pushing task and the wiping task are different, the pushing task is only conducted under the above conditions 2 and 3. The experimental steps are the same as the wiping task. The experimental results are ultimately reflected in the trajectory of the robot end effector, which determines whether the robot can complete the demonstrated task. Hence, Figure 11 only presents the trajectories of the real Baxter robot in the simulation scene and real environment, but not the results of data preprocessing.

As shown in Figure 11, the distance between the two tasks is very close. Any deviation in the trajectory of the robot end effector could result in the robot failing to complete the task. Nevertheless, we can find that the Baxter robot can complete the pushing task well from the experimental results. For this task, the results can directly reflect the performance of the two learning methods. Since the result of the robot end effector's trajectory under condition 3 is a failure to complete the pushing task, the corresponding result is not displayed in Figure 11. Also, the results for the wiping tasks are undiscussed in the next section. These results illustrate that the proposed method can not only improve the performance of TbD but also be applied in learning different tasks for the robot. It implies that the proposed method has good generalization ability.

## Discussion

The purpose of this work is to investigate the practical effect of the proposed method on robot TbD, as well as to explore the impact on the result considering the fusion of multiple modality information. It is noted that only the results of the wiping task are discussed in this section. Because the pushing task requires more accurate execution for the Baxter robot, the performance of the learning method can be directly judged by the execution results of the Baxter robot. The experimental results of the pushing task clearly illustrate that using the incremental learning method can enable the robot to complete the pushing task well, while the ELM algorithm cannot.

Firstly, we examine the effectiveness of multimodal data fusion by comparing the results in Figures 10A,B under the first and second conditions. It is clear that the bias between the reference trajectories and the real trajectories of the first condition is much larger than the second one, especially in the start phase of the interval (0, 100). And the curves of the real trajectories are inconsistent for the first and second conditions in Figures 10A,B. For joints S0 and S1, the trend of reference trajectories is almost the same under conditions 2 and 3. Concerning the joint E1, the differences between the curves are especially evident under the same conditions. In Figure 10A, the maximum difference value between the reference and the real value is already close to 2. But this value is not more than 0.6 under the second condition, which can be observed from Figure 10B. The trajectories of the real Baxter robot regarding the joints S0, S1, and E1 illustrate that the multimodal data fusion can promote a result that is much closer to the reference values.

**Figure 12:**
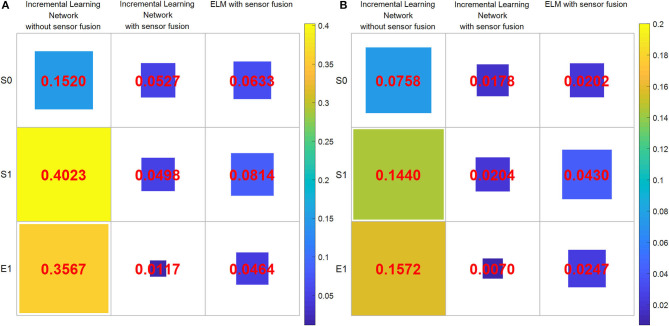
The errors of the root mean square error (RMSE) and mean absolute error (MAE) between the learned joint angles and the real Baxter robot joint values under the three experimental conditions. **(A)** RMSE. **(B)** MAE. The area of each color square indicates the magnitude of two errors.

Next, we discuss the superiority of the proposed method in comparison with another robot learning method. Figures 10B,C show the corresponding results by using the proposed method and the ELM method. The red dotted lines are the learning result of the two networks. Obviously, the trends of red reference trajectories in Figures 10B,C are consistent. It indicates that both of the methods can learn the features of the joint angles to complete the wiping task. However, the biases between the reference trajectories and the real trajectories of the two methods are different. To analyze the result quantitatively, we calculate the mean absolute error (MAE) and root mean square error (RMSE), which are shown in the second and third columns of Figures 12A,B. The RMSE is calculated as follows:

(17) $$\begin{array}{l} \text{RMSE}=\sqrt{\frac{1}{N}\overset{N}{\underset{t=1}{\sum }}{\left(y_{t}-{ŷ}_{t}\right)}^{2}}, \end{array}$$

where *N* is the size of the demonstration sequences, ŷ_*t*_ is the value fused by the KF algorithm, and *y*_*t*_ is the value measured by the Kinect sensor.

The calculation of MAE is as follows:

(18) $$\begin{array}{l} \text{MAE}=\frac{1}{N}\overset{N}{\underset{t=1}{\sum }}|y_{t}-{ŷ}_{t}|, \end{array}$$

Noteworthy, RMSE and MAE are the errors between the reference data (namely, the output of the incremental learning network or the learned trajectories) and that of the real Baxter robot data. The areas of the squares using the proposed method are less than that of squares using the ELM method. The RMSE and MAE results imply that the errors of the incremental learning method are smaller. This shows that the experiment performance of the proposed method is better than the ELM method. We can also find that the maximum error under the three conditions is from the result without data fusion. It also implies that through data fusion, both errors are diminished notably.

As aforementioned, the difficulty of the wiping task is how to ensure that the trajectories of upward and down motion are consistent. We find that the result under condition 1 is worst, and the trajectory is disordered in Figure 10D. On the contrary, the trajectories under the second and third conditions are smooth and orderly. Furthermore, the result of the second condition is better than that of the third one. It also proves that data fusion can improve the experiment performance in another way. Besides, we compute the distance between the start point and the end point for three conditions, which are 0.8825, 0.0135, and 0.0778, respectively. For the results, the shorter the distance, the better the performance. It is obvious that the distance for the second condition is the shortest. That is to say, both the trajectories of the end effector and the distance illustrate that the proposed method is better. These results suggest that the proposed method is superior to the ELM method, not only the joint angles but also the trajectories of the end effector.

Lastly, we compare all recorded joint angles of the robot's left arm under the three conditions. The desired result is that the other four joint angles are approximate to zero except for S0, S1, and E1, which is shown in Figure 10E. The four joint angles in the interval (0, 100) under the first condition are much bigger than zero, and then they gradually trend to zero. However, the four joint angles are much closer to zero under the second and third conditions from beginning to end. It also shows that sensor fusion can decrease demonstration errors.

To sum up, the demonstration data with multimodal information can significantly improve the experiment performance, and the proposed method can achieve a better execution result with smaller errors. This is probably because data fusion is beneficial to obtain a demonstration dataset close to the real value. At the same time, the KF algorithm smooths the raw data to some extent. All of these help the real robot move smoothly and efficiently. On the other hand, the incremental learning network can learn more effective features to enhance TbD performance.

## Conclusions and Future Work

In this paper, we propose an incremental learning framework to learn demonstration features by integrating different modality data. Using the proposed method and the KF algorithm, the TbD performance is remarkably improved. To verify the proposed method, comparative experiments involving the incremental learning network and ELM algorithm were conducted based on a Baxter robot in a real physical environment. Through the experiments, the robot achieved a better result with smaller errors using the proposed network on the basis of two modality information fusions. The effectiveness of the proposed method was verified by analyzing the learned data and the real robot data in comparison with ELM methods. As a result, the proposed method can learn more critical features to get the desired result. Since the TbD system is based on two modality information fusions, we also verify the effect of multimodal integration on the real robot. Compared with the results of single-modality data, the multimodal data with sensor fusion can achieve a better performance. It implies that the fusion of modality information is beneficial to improve the accuracy of data. To test the generalization of the proposed method, a pushing task is performed. The successful experiment results show that the proposed method has the generalization ability in TbD. In the future, integrating modality information from different types of sensors, e.g., force, will be addressed to perform complex tasks online. We will further explore the complete time of a specific task for the real robot by employing other methods. Also, how to reduce the effect of demonstrations from different people on the experimental results is taken into account.

## Data Availability Statement

The datasets generated for this study are available on request to the corresponding author upon reasonable request.

## Author Contributions

JL, JZ, and CY conceived of the presented idea. JL implemented the framework and conducted the experiment. JL and CY contributed to results analysis. JL contributed to manuscript writing, original draft. JZ, JY, and CY contributed to review and provide critical feedback. All authors had read the manuscript and agreed with its content.

## Conflict of Interest

The authors declare that the research was conducted in the absence of any commercial or financial relationships that could be construed as a potential conflict of interest.
